# High-pitch photon-counting detector computed tomography angiography of the coronary arteries: Qualitative and quantitative evaluation of monoenergetic image reconstructions

**DOI:** 10.1016/j.ejro.2025.100666

**Published:** 2025-06-13

**Authors:** Andreas Strassl, Francesco Lauriero, Maria Alejandra Rueda, Christian Wassipaul, Michael Weber, Christian Loewe, Dietrich Beitzke, Lucian Beer

**Affiliations:** a Division of Cardiovascular and Interventional Radiology, Department of Biomedical Imaging and Image-guided Therapy, Medical University of Vienna, Währinger Gürtel 18-20, Vienna 1090, Austria; b Christian Doppler Laboratory for Machine Learning Driven Precision Medicine, Department of Biomedical Imaging and Image-guided Therapy, Medical University Vienna, Währinger Gürtel 18-20, Vienna 1090, Austria; c Department of Diagnostic Imaging, Radiation Oncology and Hematology, Fondazione Policlinico Universitario A. Gemelli IRCCS, Largo Agostino Gemelli 8, Rome 00168, Italy; d Fundación Santa Fe de Bogotá University Hospital, Carrera 7 No. 117 −15, Bogotá DC, Colombia

**Keywords:** Coronary artery disease, Coronary CTA, Photon-counting detector CT, Contrast media, Virtual monoenergetic image

## Abstract

**Background:**

Dual-source photon-counting detector computed tomography (PCDCT) offers the opportunity to perform cardiac examinations within one beat and simultaneously the acquisition of spectral information. This study, evaluated subjective and objective image quality of virtual monoenergetic image (VMI) reconstructions using data from a first-generation, dual-source PCDCT scanner, operated in high-pitch scanning mode.

**Methods:**

We retrospectively included 30 patients who underwent a clinically indicated CTA of the coronary arteries. VMI were reconstructed at five different energy levels. Subjective image quality was assessed by three radiologists according to a four-point Likert scale for four different quality features. To evaluate objective image quality, SNR and CNR were calculated via ROIs placed in the aorta, coronary arteries, myocardium, pectoral muscle, and epicardial fat.

**Results:**

VMI at 40, 50, 60, and 70 keV showed equal mean scores (4/4) for subjective vascular contrast, followed by 80 keV reconstructions with a mean score of 3/4. The 40 keV reconstruction yielded the lowest range (3−4) in Likert scores and highest percentage of reader agreement (80 %). Minor differences in subjective image noise, sharpness, and plaque visualization were observed with positive trends toward higher keV levels. SNR and CNR were superior for 40 keV, with a mean of 34.8 ± 1.7HU and 45.4 ± 2.7HU, respectively. Mean applied contrast volume was 65 ml, resulting in a mean CT value of 1150HU for 40 keV VMI.

**Conclusion:**

First-generation PCDCT-derived VMI at 40 and 50 keV offer satisfying subjective and objective image quality, even when acquired in high-pitch scanning mode.

## Highlights

• Photon-counting detector computed tomography enables virtual monoenergetic images at various keV levels.
• Virtual monoenergetic images in cardiac computed tomography enable improved vascular contrast.
• Photon Counting derived virtual monoenergetic images at 40 and 50 keV offer satisfying image quality, at high-pitch scanning mode.

## 1 Introduction

Photon-counting detector computed tomography (PCDCT) scanners are the most recent CT scanner generation. In contrast to conventional energy-integrating detector CT (EIDCT) systems, PCDCT scanners use a different physical principle in detector technology as they directly convert incident x-ray photons into electrical signals without the need for conversion into visible light prior to signal induction. While an EIDCT sums and “integrates” the total energy spectrum of all photons that reach the detector, PCDCT is, in theory, able to directly measure the energy of a single photon. Through the establishment of a minimum threshold at a level higher than the signal intensity of electronic noise, it is possible to reduce image noise, even at low radiation doses. By introducing thresholds at different energy levels, photons can be sorted into distinct energy bins, allowing for energy discrimination and, consequently, spectral imaging [1], [2], [3]. All aforementioned physical and technical characteristics give PCDCT multiple advantages over EIDCT: improved spatial resolution; lower radiation dose; image noise reduction; and acquisition of spectral information. These properties, consequently, allow contrast-to-noise improvement, material decomposition, and artifact reduction. Therefore, PCDCT offers the potential to improve the clinical use of CT imaging and to add new applications [3], [4].

To date, dual-source CT scanners with high-pitch scanning modes are frequently used for coronary CT angiography to rule out coronary artery disease (CAD), allowing motion-free imaging of the coronary arteries at low radiation and iodine doses [5]. However, VMI, already known from dual-energy CT (DECT) imaging, has the potential to further reduce contrast agent doses due to an increase of contrast-to-noise ratios (CNR) at low kilo-electron-volt (keV) levels, at the cost of reduced temporal resolution in dual-source DECT and overall increased image noise [6]. Until the introduction of the first clinical PCDCT, the combination of both high-pitch scanning and spectral imaging was not possible within one examination. The advent of dual-source PCDCT has the potential to significantly improve the radiological evaluation of coronary arteries, and therefore, the assessment of CAD [7], as initial studies on patients have already shown improved morphologic visualization of vessels in other body regions [8], [9], [10], [11], [12].

### 1.1 Study aim

In this analysis, the image quality and detectability of coronary arteries have been evaluated using VMI reconstructions with five different keV levels using PCDCT and a high-pitch scanning mode to evaluate CAD. The acquired results shall provide a basis for optimized image reconstructions for CCTA with the long-term aim to enable a reduction in the iodine dose patients receive.

## 2 Methods

### 2.1 Study population

We retrospectively evaluated patients who underwent a clinically indicated CCTA of the coronary arteries, for the work-up of chronic coronary syndrome. Exams were performed at the Department of Biomedical Imaging and Image-guided Therapy, Medical University of Vienna, between April and June 2022. The study was approved by the institutional review board (EK Nr. 1652/2022). In addition, written, informed consent for processing image and clinical data was obtained from all patients before the examination in the course of the PCDCT registry study (EK Nr. 2032/2021). Participants were consecutively selected according to the defined inclusion and exclusion criteria. Since this is a pilot study, no sample size calculation was executed.

**Fig. 1:**
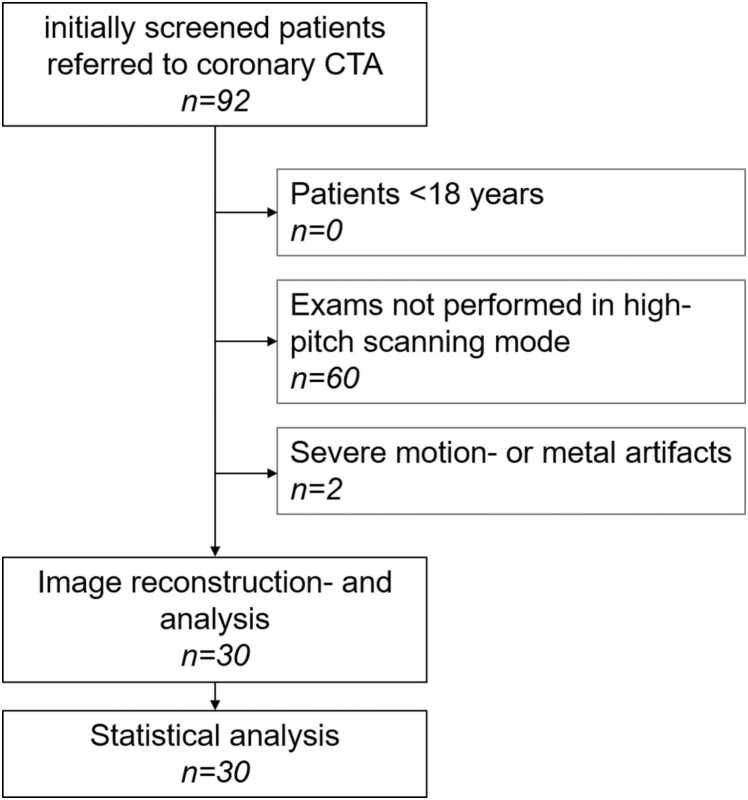
Flowchart of study inclusion and exclusion.

Inclusion criteria were as follows: age > 18 years; clinical indication for a coronary CTA scan; written informed consent before the examination; data acquired with a high-pitch prospective ECG-triggered scanning-mode. Patients were excluded in case of severe motion or metal artifacts (Fig. 1). This study was conducted in accordance with the Declaration of Helsinki.

### 2.2 Image acquisition

All examinations were performed on a Siemens NAEOTOM Alpha PCDCT scanner (Siemens Healthineers, Forchheim, Germany) using the institutional standard imaging and contrast media injection protocol for coronary CTA (Visipaque 320 mg/ml, GE Healthcare Handels GmbH, Vienna, Austria; 62 ml, flowrate 4.1 ml/s and bolus-tracking technique for scan-timing with an attenuation threshold of 100HU in the ascending aorta). Contrast volume and flow were adjusted by technicians for patients with distinctly deviating body weight. The imaging protocol also included a coronary artery calcium (CAC) scan prior to CTA in order to calculate Agatston score. Intravenous beta-blockers were applied in patients with elevated heart rates (>80bpm).

### 2.3 Image reconstruction and analysis

For each patient, the archived spectral CT image data set was used to compute five different monoenergetic image series (40, 50, 60, 70, and 80 keV) using a dedicated CT postprocessing software (SyngoVia VB60 CT Dual Energy, Siemens Healthineers, Forchheim, Germany). Agatston score was computed in accordance to international standards (SyngoVia VB60 CT CaScoring, Siemens Healthineers, Forchheim, Germany). Acquisition and reconstruction parameters can be found in Table 1.

**Table 1:** Scan and reconstruction parameters.

**Scan Parameters**	*CTA*	*CAC scan*
Collimation	144 × 0.4 mm	144 × 0.4 mm
Tube potential	120 kVp	120 kVp
CARE keV IQ level	90	19
Rotation time	0.25 s	0.25 s
Pitch factor	3.2	3.2
**Reconstruction Parameters**	*Setting*	*CAC scan*
Image type	axial MPR	fixed axial
Reconstructed slice thickness	0.4 mm	3.0 mm
Reconstruction increment	0.3 mm	1.5 mm
Kernel	Bv40	Qr36
Matrix size	512 × 512	512 × 512
Reconstructed field of view	limited to the heart	limited to the heart
Quantum Iterative Reconstruction (QIR)	Level 3	off
Cardiac phase	65 %	75 %
IQ=Image Quality; MPR= Multiplanar Reformation		

For subjective image quality assessment, all image reconstructions were pseudonymized and loaded and separately displayed on the viewing workstation (SyngoVia VB60 MM Reading, Siemens Healthineers, Forchheim, Germany). For image quality assessment, three radiologists with different levels of expertise in cardiac imaging (one radiologist with five years’ experience and two radiologists with one year in cardiac radiology) independently evaluated the randomly assigned images using established quality criteria for cardiac imaging. The readers were blinded to the applied keV level of the VMI reconstruction. For each review, the readers were asked to assess four image quality features according to a four-point Likert scale, already used in previous studies: image-noise [13]; image-sharpness [14]; vascular contrast [15]; and depiction of calcified coronary plaques [16], see Table 2. Window settings were freely adjustable by the readers.

**Table 2:** Image quality criteria for subjective evaluation.

Image Quality Classification	
*Definition*	*Rating*
**Image Noise**	
not diagnostic	1
above average noise	2
average noise	3
no noise	4
**Image Sharpness**	
severe artifacts with inadequate delineation between the lumen and the surrounding tissue	1
adequate image quality (noticeably blurred vessel, but acceptable for diagnosis)	2
good image quality (blurring of vessel margin and minor artifacts, fully evaluable)	3
excellent image quality (with the absence of artifacts)	4
**Vascular Contrast**	
no enhancement of one or more vessels, no diagnosis possible	1
slight to moderate enhancement, sufficient for diagnosis	2
good enhancement	3
excellent enhancement	4
**Coronary Plaque Morphology**	
not applicable due to low plaque volume	0
low	1
adequate	2
good	3
optimal	4

For quantitative image quality assessment, a CT technologist (A.S.) measured mean CT numbers and standard deviation (SD) of multiple regions-of-interest (ROI) to calculate SNR as *Mean HU/SD* and CNR *(Mean HU*_*vessel*_ *– Mean HU*_*background*_*)/SD*_*background*_. The evaluation was performed using the same clinically available viewing workstation. To reduce measurement inaccuracies due to ROI positioning, all calculations were made twice and the average of the obtained values was calculated. Since 70 keV is set as the standard keV level for many image reconstructions and HU values closely correspond to standard polychromatic 120 kVp EID-CT images,^10^ differences of means between 70 and 40/50/60/80 keV were used for statistical analysis, alongside absolute values. Due to the retrospective nature of the study and consequently the absence of CT raw data at the time of evaluation, conventional image reconstructions were not available for comparison. Detailed information about the measurements is provided in Table 3.

**Table 3:** ROI positioning and sizing for quantitative evaluation.

Image Measurements		
*ROI*	*Size [mm*^*2*^*]*	*Position*
Aorta	2500	ascending aorta at the level of LM ostium
RCA	> 1	Segment 1*
LAD	> 1	Segment 6*
LCX	> 1	Segment 11*
Muscle	200	left pectoralis major muscle
Myocardium	200	interventricular septum
Epicardial fat	200	below the level of RCA ostium

LM = Left Main Artery; RCA = Right Coronary Artery; LAD = Left Anterior Descending Artery; LCX = Left Circumflex Artery

* according to the Society of Cardiovascular Computed Tomography (SCCT) coronary segmentation diagram

### 2.4 Statistical analysis

All data were analyzed using IBM SPSS for Windows version 28 (IBM Corp., Armonk NY, USA). Metric and normally distributed data are described using the mean ± SD. Skewed metric data and ordinal data are described using median [min; max] and IQR when appropriate. Barcharts and boxplots were used for graphical presentation of the results. Repeated measures ANOVAs were used to compare the average of metric data (SNR, CNR, HU) between different keV levels. As repeated measures ANOVAs only assume normal distributed differences and are well known to be robust against violation of this assumption, we did not test for normal distributed differences. In addition, Bonferroni-corrected contrasts were used to compare the average of 70 keV images to all other keV levels. P-values equal to or below 0.05 were considered statistically significant. Due to the small sample size, no multiplicity corrections were performed to avoid an increased error of the second type. To assess rater agreement, the percentage of cases where all raters rated equally was calculated.

## 3 Results

The study population consisted of 30 patients with a mean age of 60 ± 15 years and a body-mass-index of 26.2 ± 5.3. The gender ratio was 1:1, although there was no specific patient selection. The mean applied contrast volume was 65 ± 9 ml, using a flow-rate of 4.1 ± 0.5 ml/s. Mean dose-length-product (DLP) for the CTA scan only was 67 ± 20mGycm. Coronary plaque burden, effective lumen diameters of coronary arteries, and reconstruction details are summarized in Table 4.

**Table 4:** Patient- and exam-related characteristics.

	Mean	SD	Median	Minimum	Maximum	IQR
Age	60	15	59	24	82	20
Height [cm]	1,7	0,1	1,7	1,5	1,9	0,2
Weight [kg]	77	16	80	41	106	25
BMI	26,2	5,3	26,6	14,2	34,9	7,4
Contrast volume [ml]	65	9	66	45	80	15
Contrast flowrate [ml/s]	4,1	0,5	4,4	3,0	5,0	0,8
Heart rate [1/min]	59	8	57	38	75	10
CTDI_vol_ [mGy]	3,6	1,1	3,6	1,8	6,3	1,3
DLP CTA [mGycm]	67	20	66	33	125	25
DLP total [mGycm]	93	27	97	46	169	38
N. o. images	368	36	370	294	440	54
FOV [mm]	169	14	170	144	200	22
In-Plane resolution [mm]	0,33	0,03	0,33	0,28	0,39	0,04
Agatston score	223	418	53	0	2103	291
Diameter* RCA [mm]	3,0	0,7	3,0	1,7	4,8	1,0
Diameter* LAD [mm]	2,6	0,7	2,3	1,6	4,1	0,8
Diameter* LCX [mm]	2,6	0,6	2,6	1,8	4,1	0,9

SD = Standard Deviation; IQR = Interquartile Range; BMI = Body Mass Index;

CTDI_vol_ = Volumetric Computed Tomography Dose Index; DLP = Dose Length Product; FOV = Field Of View

Total DLP includes scout- and bolus tracking scans, coronary calcium scoring scan and CTA

* effective diameter $D_{\mathrm{eff}}=2\;\times \;\left|\sqrt{\frac{A}{\pi }}\right|$

According to the classification by Gupta et al. [17], there were eight patients without, seven with mild, seven with moderate, and five patients with severe coronary artery calcification in the study cohort, while the Agatston score was missing in three patients.

### 3.1 Subjective image quality

#### 3.1.1 Contrast

VMI showed excellent vessel contrast for 40, 50, 60, and 70 keV (median of 4; pooled for all readers) and good contrast for 80 keV (median 3; range 2–4) reconstructions. Best results were achieved for 40 keV (median 4; range 3–4), with the highest minimum score. In addition, the 40 keV reconstruction showed the best inter-reader agreement, with 80 %, followed by 50 keV and 60 keV with 77 % and 67 %, respectively.

#### 3.1.2 Sharpness

Vessel delineation was good (median 3) for all VMI reconstructions; however, the 40 keV reconstruction showed the widest range (1−4) with the lowest minimum score and was therefore slightly inferior compared to all other energy levels (range 2–4). The 40 keV reconstruction also showed a poor inter-reader agreement of 17 % versus > 33 % for 50, 60, 70, and 80 keV.

#### 3.1.3 Noise

Image noise was rated as average (median 3; range 1–4) and therefore also comparable for all VMI reconstructions with a trend toward higher energy levels in terms of noise reduction. However, this trend was accompanied by lower inter-reader agreement (10–13 %).

#### 3.1.4 Plaques

Depiction of coronary plaque morphology was good (median of 3; range 1–4), and no tendencies toward any keV level were observed. Inter-reader agreement was similarly poor for all energy levels (14–19 %). Since some patients had an Agatston score of ≤ 1 (n = 9), the total count of ratings for plaque visibility was fewer than for the other image quality criteria. In patients with Agatston score > 1 but very low plaque volume, a rating of zero was defined as “not applicable” to assess this image quality feature.

**Fig. 2:**
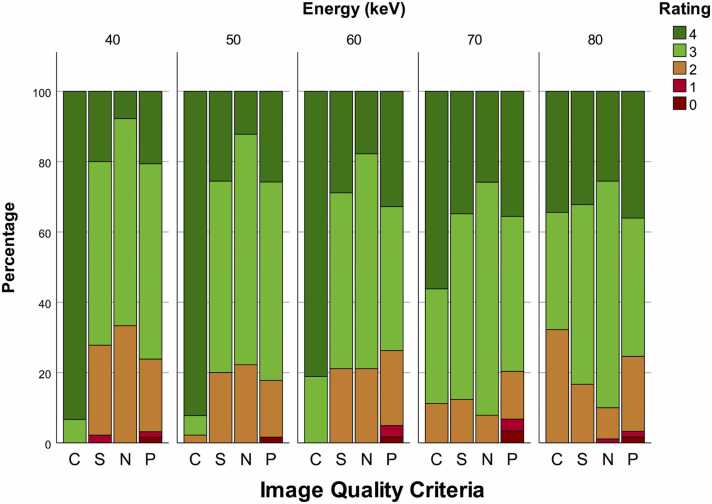
Pooled scores for vessel contrast (C), image-sharpness (S), image-noise (N), and plaque morphology (P) at different energies.

**Fig. 3:**
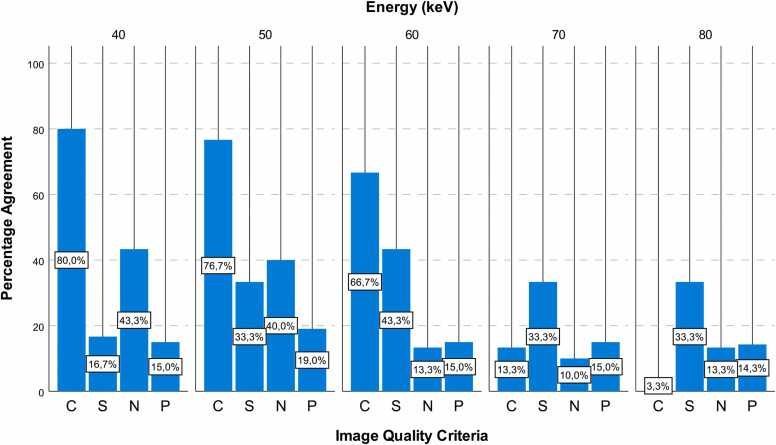
Percentage reader agreement for vessel contrast (C), image-sharpness (S), image-noise (N) and plaque morphology (P) at different energies.

Fig. 2 shows pooled scores for all readers. Percentage reader-agreement is shown in Fig. 3.

### 3.2 Objective image quality

All quantitative image quality data were normally distributed. CT numbers measured in the aortic ROIs are shown in brackets, unless otherwise described.

#### 3.2.1 Attenuation

The average vascular attenuation showed a significant increase with decreasing keV levels (mean ± std. error; *p* < 0.001 for all):

• 80 keV: 306 ± 14 HU
• 70 keV: 397 ± 15 HU
• 60 keV: 539 ± 18 HU
• 50 keV: 770 ± 25 HU
• 40 keV: 1150 ± 34 HU

The highest absolute HU values were observed for 40 keV (1150 ± 34; 1083–1217, mean ± std. error; 95 % CI) in all regions except epicardial fat. For epicardial fat, 80 keV reconstructions showed the highest CT numbers (-78 ± 2; −82 to −73).

#### 3.2.2 Noise and SNR

The highest image noise was observed for 40 keV (34 ± 1; 32–37) in all regions; however, SNR was superior in 40 keV reconstructions (35 ± 2; 31–38) for all ROIs placed in vascular structures (Aorta, RCA, LAD, and LCX). For pectoral muscle and epicardial fat, 80 keV yielded the highest SNR (4.7 ± 0.3; 4.1–5.5 and 5.2 ± 0.3; 4.6–5.8) and for myocardium, 70 keV (6.2 ± 0.3; 5.7–6.7).

#### 3.2.3 Contrast

**Fig. 4:**
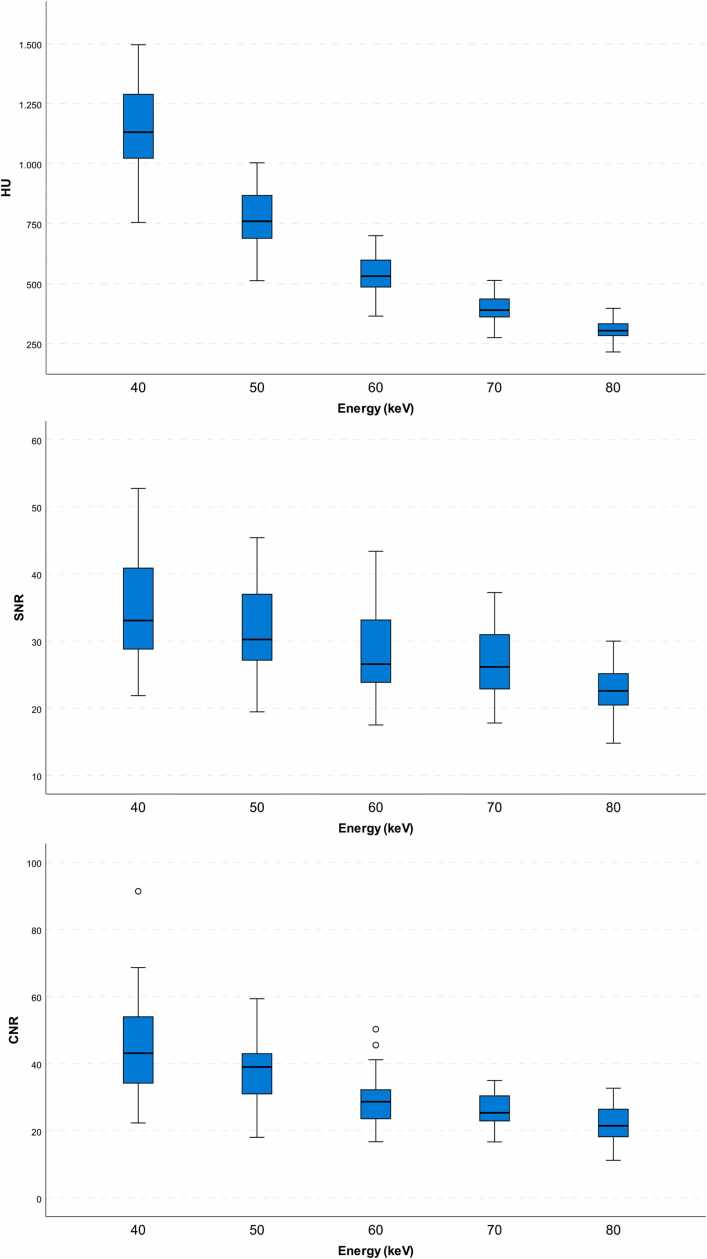
Boxplots for absolute HU, SNR, and CNR measured in the ascending aorta at different energies.

The highest CNR values for all vascular structures were measured for 40 keV, regardless of whether the CT numbers for pectoral muscle (45 ± 3; 40–51), myocardium (32 ± 1; 30–34), or epicardial fat (40 ± 3; 34–45) were used as a background value for CNR calculation. Fig. 4 shows absolute HU, SNR, and CNR for aortic ROIs for all keV levels.

Absolute differences (ΔHU) between 70 keV and 40/50/60 or 80 keV showed statistically significant (*p* < 0.001) changes for all keV levels in all vascular structures. Also, image noise differed significantly between 70 keV and 40/50/60 or 80 keV (*p* < 0.004). For ΔSNR, statistical significance could be shown between 70 and 40/50 keV in all vessels (*p* < 0.004) except the RCA (*p* = 0.473) for 70 keV vs. 50 keV. For ΔCNR, statistically significant differences were observed for all vessels across all keV levels (*p* < 0.013), maximum differences to 70 keV were observed in 40 keV VMI (753 ± 23HU; mean ± std. error).

## 4 Discussion

In this study, we evaluated the image quality of virtual monoenergetic image (VMI) reconstructions using data acquired with a high-pitch scanning mode on a first-generation PCDCT scanner. Vascular contrast was superior with 40 keV reconstructions when evaluating subjective image quality and also showed the highest CNR, as a surrogate for quantitative evaluation. Although 40 keV VMI showed the highest image noise, SNR was superior due to the substantially increased iodine signal. Subjective image quality assessment yielded almost similar results for 40 and 50 keV in terms of vascular contrast, but with higher scores and better reader agreement for image noise, sharpness, and plaque morphology for 50 keV. Despite the slightly superior ‘numerical image quality’ at 40 keV, VMI at 50 keV seems to ensure a better trade-off for overall subjective image quality. VMI with even higher keV levels offer less image noise and vessel blurring, as well as improved depiction of plaque morphology, but, at the cost of inferior vascular contrast.

Up until now, the majority of the PCDCT studies were performed on prototype scanners in a research environment with small sample sizes. Regarding imaging of CAD, the authors focused on ultra-high-resolution (UHR) image acquisitions, stenosis quantification or compared their results with data from EIDCT scanners [18], [19], [20]. Therefore, to the best of our knowledge, this is one of the first studies to compare different VMI reconstructions of coronary arteries, myocardium, and perivascular fat, using an electrocardiogram-triggered high-pitch scanning mode.

**Fig. 5:**
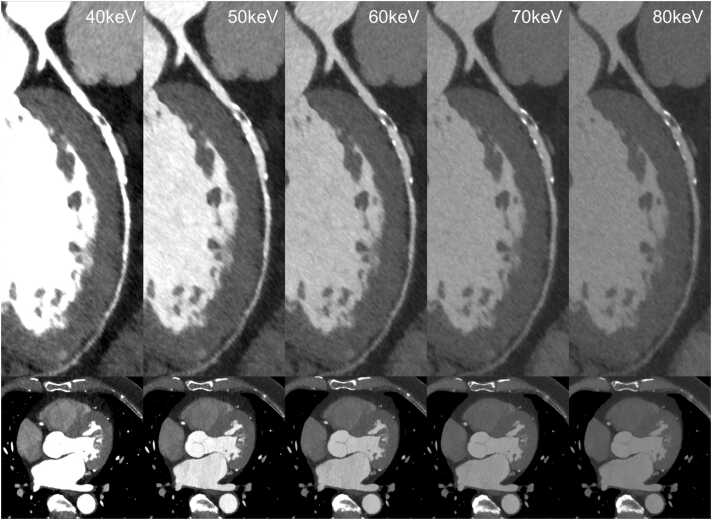
Curved planar reconstructions (CPRs) of the LAD and axial MPRs of the heart at the aortic valve level showing a significant graded increase of vascular attenuation with decreasing kev levels.

This analysis shows that SNR and CNR increase with decreasing keV levels (Fig. 5); however, the increase of SNR and CNR is lower compared to that of absolute CT numbers. Even so, reduced image quality at lower keV levels is no longer a concern, since SNR overall is high. Prior generations of VMI computation algorithms using DECT were associated with increased image noise: Arendt et al. did a comprehensive evaluation of image quality for the VMI basic algorithm and VMI+ , the development of the basic algorithm [6]. They showed markedly reduced image noise for VMI+ , resulting in increased SNR (44.5) and CNR (33.5) in 40 keV images. PCDCT, so far, has shown promising results concerning image noise reduction. The amplitude of the current pulses produced by absorbed photons needs to exceed a threshold of 25 keV to trigger a count; thus, baseline noise caused by low-energy x-rays can effectively be reduced [1]. With PCDCT-derived VMI, we therefore achieved higher SNR (46.9) and CNR (35.7) for the LAD, regardless of scan parameters and radiation dose, even at thinner slices (0.40 mm vs. 0.75 mm).

Spectral imaging of the heart and coronary arteries at low radiation doses may become one of the major benefits of PCDCT imaging. Although the use of the VMI technique for cardiac imaging is not completely new, not having to compromise on scan duration is another benefit of PCDCT compared to DECT (scan duration of 300 ms vs. 7000 ms). Ahmed et al. used a cardiac motion phantom to demonstrate the geometric accuracy and motion robustness of material decomposition images performed with PCDCT and dual-source DECT. They showed that, with high-pitch dual-source PCDCT, excellent geometric accuracy and robustness at similar image noise can be achieved while reducing radiation dose by 62 %. In addition, DECT is more sensitive to motion artifacts due to lower temporal resolution [21]. Although the computation of material decomposed images relies on different algorithms, compared to VMI, the consequences of geometrical uncertainties apply for both image types.

In this study, inter-reader agreement for vascular contrast was good, underlining the excellent CNR of 40 and 50 keV VMI. For all other subjective quality criteria, agreement was rather poor. Comparing the individual results of the three different readers, it was observed that they had a different subjective perception of the individual scores. However, the total count per reader systematically tended toward higher or lower values. Notably, subjective evaluation of coronary plaque morphology showed poor inter-reader agreement for all keV-levels. This may be attributed to the smaller sample size for patients with plaques (n = 19), rather unspecific definitions for the different plaque-scores compared to the other image quality criteria, and the low overall plaque burden with a median Agatston score of 53.

Absolute HU, noise, SNR, and CNR measured in the coronary arteries basically correspond to those values measured in the aortic root, presented in the Results section. The lower image noise measured for the LAD and LCX was perhaps caused by smaller ROI diameters. In addition, less coronary artery motion has been reported for the LAD and LCX compared to the RCA [22]. Our results for the aortic regions are in line with those reported by Euler et al., who compared different VMI reconstructions for high-pitch CTA acquisition of the aorta. They found 45–50 keV to be a good trade-off between subjective and objective image quality [8].

Interestingly, the mean attenuation for epicardial fat was lowest for 40 keV and highest for 80 keV, inversely to pectoral muscle and myocardium. This is perhaps caused by the different tissue composition and reduced iodine uptake of epicardial fat compared to muscle, even in an early-contrast phase. The same relations were found by Vattay et al. who compared VMI from 50 to 80 keV and virtual polychromatic images (T3D), the latter correlating closely with 70 keV VMI. There was a difference of −18.5HU between 50 keV VMI and T3D, and the lowest variation was observed between 70 keV VMI and T3D with −1.3HU (absolute −79.6 ± 7.8 HU) [23]. Their absolute HU-range approximately agrees with our data (-83,0 ± 3.5 HU) for 70 keV, but shows higher magnitude for 50 keV versus 70 keV, with −37.9HU, and even higher for 40 keV versus 70 keV, with −63.5HU. However, SNR for epicardial fat was superior for 80 keV (5.2 ± 0.3) in our cohort, as well as for the pectoral muscle (4.8 ± 0.3) and for myocardium at 70 keV (6.2 ± 0.3). Despite the high difference between vessel and perivascular fat attenuation at 40 keV, no higher CNR values can be measured when placing ROIs in epicardial fat due to increased image noise, and slightly reduced SNR at low keV levels. Since perivascular fat attenuation as an indirect measure of adipocyte size and lipid content, reflecting inflammation, is becoming increasingly important, this conclusions may be of particular interest [24]. Similar to coronary artery calcium scoring, where tube-voltage needs to be fixed, the keV level is an issue in PCDCT scanning to achieve standardization of measurements. A whole range of imaging parameters that could potentially exert an influence on coronary plaque volume have been assessed already; also for PCDCT-derived VMI, energy level dependance has been shown: mean plaque volume decreases with increasing energy level [25]. This seems reasonable since higher keV images decrease blooming artifacts and image noise, enhancing the analysis of calcified plaques. In our analysis, there was also a trend toward higher keV levels for subjective image quality; however, the number of ratings for adequate or low image quality were comparable between all energy levels.

The aim of this study was to provide data as a basis for future investigations that will consequently result in a direct benefit for patients, particularly in terms of contrast media reduction. Due to an average delta of 601 HU for 40 keV and 279HU for 50 keV, and considering an optimized enhancement at 400HU [26], substantial reduction of iodine dose may be achieved, enabling a safe contrast administration, especially in vulnerable patient groups.

Our results are in line with Cundari et al. [27], who showed that PCDCT-derived VMI for CCTA, at an optimized VMI level of 45 keV, allows for a contrast media reduction of 40 %. For the non-reduced (iodine concentration 370 mg/ml; volume 72–85,2 ml) patient cohort, their results show good agreement for objective and subjective image quality, mentioning that the authors focused on image contrast and noise only, but used overall image quality for the selection of the most applicable VMI keV-level. In addition, a sequential scan-mode was used for spectral image acquisition.

Rajiah et al. used a contrast media protocol reduced by 50 %, resulting in 30 ml contrast agent volume with an iodine concentration of 250 mg/ml, using a high-pitch scanning mode. They found 50 keV to be safe for use in the clinical routine and recommended 100 keV VMI to improve visualization of residual coronary artery lumen caused by calcified plaques and stents. However, the readers assigned a CAD-RADS score and compared it to a previous scoring without VMI available and the score was re-categorized in nine of 27 cases, underlining the clinical relevance of utilizing PCDCT-derived VMI. Notably, they evaluated image quality for only 50 and 100 keV VMI and T3D images [28].

Another aspect related to the optimal VMI level is that PCDCT imaging produces more CT data by orders of magnitude due to increased spatial resolution in-plane, as well as in the Z-direction (0.4 mm) and offers the opportunity to reconstruct various VMI and other spectral images, such as Virtual-Non-Iodine (VNI) and Virtual-Non-Calcium (VNCa) maps. Hence, in clinical routine, a well-considered selection of the most useful image reconstructions for the diagnosis of CAD is necessary to avoid a significant increase of CT data volume, which would result in longer reconstruction- and transfer-times, higher image-storage capacity requirements, and longer reporting times.

### 4.1 Limitations

This analysis does not include CCTA exams acquired with a sequential or standard-pitch helical scan-mode, which are routinely performed in patients with tachycardia or arrhythmia. On the scanner used, automatic selection of the cardiac scan protocol is performed for each individual, depending on heart rate, heart rate variability, and patient weight (institutional cut-off 100 kg). The workflow presented here leads to an optimized patient cohort that enables high-pitch acquisitions but do not necessarily reflect the average patient referred to CTA of the coronaries. Another limitation is the relatively inexperience of the readers performing subjective evaluation. Since this is a pilot study, the investigated cohort is rather small. Further studies with larger patient cohorts are required in the future to verify our results.

## 5 Conclusion

First-generation PCDCT-derived VMI at 40 and 50 keV offer satisfying subjective and objective image quality for high-pitch acquisitions of the heart, therefore have the potential to further reduce patients’ iodine dose in future.

## CRediT authorship contribution statement

**Dietrich Beitzke:** Writing – review & editing, Writing – original draft, Validation, Supervision, Methodology, Investigation, Data curation. **Christian Loewe:** Writing – review & editing, Supervision, Software, Resources, Conceptualization. **Michael Weber:** Writing – review & editing, Validation, Software, Formal analysis, Data curation. **Christian Wassipaul:** Writing – review & editing, Supervision, Software, Investigation, Data curation. **Maria Alejandra Rueda:** Writing – review & editing, Software, Methodology, Formal analysis, Data curation. **Francesco Lauriero:** Writing – review & editing, Supervision, Software, Methodology, Formal analysis, Conceptualization. **Andreas Strassl:** Writing – review & editing, Writing – original draft, Validation, Methodology, Investigation, Formal analysis, Conceptualization. **Lucian Beer:** Writing – review & editing, Writing – original draft, Visualization, Validation, Investigation, Formal analysis, Conceptualization.

## Ethics approval and consent to participate

The ethics committee of the Medical University of Vienna approved this study (EK Nr. 2032/2021). Informed consent was obtained from all participants.

## Declaration of generative AI and AI-Assisted technologies in the writing process

During the preparation of this work, the authors did not use any AI-assisted tools or services. The authors take full responsibility for the content of the publication

## Funding

This work was support by the Austrian Federal Ministry for Labour and Economy, the National Foundation for Research, Technology and Development, and the 10.13039/501100006012Christian Doppler Research Association is gratefully acknowledged.

## Declaration of Competing Interest

The authors declare the following financial interests/personal relationships which may be considered as potential competing interests: Andreas Strassl reports peakers’ honoraria from Siemens Healthineers GmbH and GE Healthcare Handels GmbH. Beitzke Dietrich reports a paid expert testimony for Siemens Heathineers, Pfizer, Bayer Austria and Medis Medical Imaging. Christian Loewe reports paid expert testimony for Siemens Heathineers If there are other authors, they declare that they have no known competing financial interests or personal relationships that could have appeared to influence the work reported in this paper.

## References

[1] Flohr T., Petersilka M., Henning A., Ulzheimer S., Ferda J., Schmidt B. (2020). Photon-counting CT review, phys. medica PM int. J. devoted. Appl. Phys. Med. Biol. Off. J. Ital. Assoc. Biomed. Phys. AIFB.

[2] Flohr T., Ulzheimer S., Petersilka M., Schmidt B. (2020). Basic principles and clinical potential of photon-counting detector CT. Chin. J. Acad. Radio..

[3] Hsieh S.S., Leng S., Rajendran K., Tao S., McCollough C.H. (2021). Photon counting CT: clinical applications and future developments. IEEE Trans. Radiat. Plasma Med. Sci..

[4] Rajendran K., Petersilka M., Henning A., Shanblatt E., Marsh J., Thorne J., Schmidt B., Flohr T., Fletcher J., McCollough C., Leng S. (2021). Full field-of-view, high-resolution, photon-counting detector CT: technical assessment and initial patient experience. Phys. Med. Biol..

[5] Zhang L.J., Qi L., De Cecco C.N., Zhou C.S., Spearman J.V., Schoepf U.J., Lu G.M. (2014). High-pitch coronary CT angiography at 70 kvp with low contrast medium volume: comparison of 80 and 100 kvp high-pitch protocols. Medicine.

[6] Arendt C.T., Czwikla R., Lenga L., Wichmann J.L., Albrecht M.H., Booz C., Martin S.S., Leithner D., Tischendorf P., Blandino A., Vogl T.J. (2020). T. D’Angelo, Improved coronary artery contrast enhancement using noise-optimised virtual monoenergetic imaging from dual-source dual-energy computed tomography. Eur. J. Radio..

[7] Flohr T., Schmidt B., Ulzheimer S., Alkadhi H. (2023). Cardiac imaging with photon counting CT. Br. J. Radio..

[8] Euler A., Higashigaito K., Mergen V., Sartoretti T., Zanini B., Schmidt B., Flohr T.G., Ulzheimer S., Eberhard M., Alkadhi H. (2022). High-pitch photon-counting detector computed tomography angiography of the aorta: intraindividual comparison to Energy-Integrating detector computed tomography at equal radiation dose. Invest. Radio..

[9] Dillinger D., Overhoff D., Booz C., Kaatsch H.L., Piechotka J., Hagen A., Froelich M.F., Vogl T.J., Waldeck S. (2023). Impact of CT photon-counting virtual monoenergetic imaging on visualization of abdominal arterial vessels. Diagnostics.

[10] Yalynska T., Polacin M., Frauenfelder T., Martini K. (2022). Impact of photon counting detector CT derived virtual monoenergetic images on the diagnosis of pulmonary embolism. Diagnostics.

[11] Symons R., Reich D.S., Bagheri M., Cork T.E., Krauss B., Ulzheimer S., Kappler S., Bluemke D.A., Pourmorteza A. (2018). Photon-counting CT for vascular imaging of the head and neck: first in vivo human results. Invest. Radio..

[12] Michael A.E., Boriesosdick J., Schoenbeck D., Woeltjen M.M., Saeed S., Kroeger J.R., Horstmeier S., Lennartz S., Borggrefe J., Niehoff J.H. (2022). Image-Quality assessment of polyenergetic and virtual monoenergetic reconstructions of unenhanced CT scans of the head: initial experiences with the first Photon-Counting CT approved for clinical use. Diagnostics.

[13] Károlyi M., Szilveszter B., Kolossváry M., Takx R.A.P., Celeng C., Bartykowszki A., Jermendy Á.L., Panajotu A., Karády J., Raaijmakers R., Giepmans W., Merkely B., Maurovich-Horvat P. (2017). Iterative model reconstruction reduces calcified plaque volume in coronary CT angiography. Eur. J. Radio..

[14] Rajiah P., Ciancibello L., Novak R., Sposato J., Landeras L., Gilkeson R. (2019). Ultra-low dose contrast CT pulmonary angiography in oncology patients using a high-pitch helical dual-source technology. Diagn. Interv. Radio..

[15] Vecsey-Nagy M., Jermendy Á.L., Suhai F.I., Panajotu A., Csőre J., Borzsák S., Fontanini D.M., Kolossváry M., Vattay B., Boussoussou M., Csobay-Novák C., Merkely B., Maurovich-Horvat P., Szilveszter B. (2021). Model-based adaptive filter for a dedicated cardiovascular CT scanner: assessment of image noise, sharpness and quality. Eur. J. Radio..

[16] Maffei E., Martini C., Arcadi T., Clemente A., Seitun S., Zuccarelli A., Torri T., Mollet N.R., Rossi A., Catalano O., Messalli G. (2012). F. Cademartiri, plaque imaging with CT coronary angiography: effect of intra-vascular attenuation on plaque type classification. World J. Radio..

[17] Gupta A., Bera K., Kikano E., Pierce J.D., Gan J., Rajdev M., Ciancibello L.M., Gupta A., Rajagopalan S., Gilkeson R.C. (2022). Coronary artery calcium scoring: current status and future directions. RadioGraphics.

[18] Greffier J., Si-Mohamed S.A., Lacombe H., Labour J., Djabli D., Boccalini S., Varasteh M., Villien M., Yagil Y., Erhard K., Boussel L., Beregi J.-P., Douek P.C. (2023). Virtual monochromatic images for coronary artery imaging with a spectral photon-counting CT in comparison to dual-layer CT systems: a phantom and a preliminary human study. Eur. Radio..

[19] Wolf E.V., Halfmann M.C., Varga-Szemes A., Fink N., Kloeckner R., Bockius S., Allmendinger T., Hagenauer J., Koehler T., Kreitner K.-F., Schoepf U.J., Münzel T., Düber C., Gori T., Yang Y., Hell M.M., Emrich T. (2024). Photon-Counting detector CT virtual monoenergetic images for coronary artery stenosis quantification: phantom and in vivo evaluation. Am. J. Roentgenol..

[20] Si-Mohamed S.A., Boccalini S., Lacombe H., Diaw A., Varasteh M., Rodesch P.-A., Dessouky R., Villien M., Tatard-Leitman V., Bochaton T., Coulon P., Yagil Y., Lahoud E., Erhard K., Riche B., Bonnefoy E., Rioufol G., Finet G., Bergerot C., Boussel L., Greffier J., Douek P.C. (2022). Coronary CT angiography with Photon-counting CT: First-In-Human results. Radiology.

[21] Ahmed Z., Campeau D., Gong H., Rajendran K., Rajiah P., McCollough C., Leng S. (2023). High-pitch, high temporal resolution, multi-energy cardiac imaging on a dual-source photon-counting-detector CT. Med. Phys..

[22] Shechter G., Resar J.R., McVeigh E.R. (2006). Displacement and velocity of the coronary arteries: cardiac and respiratory motion. IEEE Trans. Med. Imaging.

[23] Vattay B., Boussoussou M., Bartykowszki A., Kolossváry M., Konkoly G., Vecsey-Nagy M., Kubovje A., Merkely B., Maurovich-Horvat P., Szilveszter B. (2022). 428 the impact of virtual monoenergetic image energy levels on pericoronary adipose tissue attenuation using dual-source photon-counting detector computed tomography. J. Cardiovasc. Comput. Tomogr..

[24] Ma R., Fari R., van der Harst P., N. De Cecco C., E.Stillman A., Vliegenthart R., van Assen M. (2023). Evaluation of pericoronary adipose tissue attenuation on CT. Br. J. Radio..

[25] Vattay B., Szilveszter B., Boussoussou M., Vecsey-Nagy M., Lin A., Konkoly G., Kubovje A., Schwarz F., Merkely B., Maurovich-Horvat P., Williams M.C., Dey D., Kolossváry M. (2023). Impact of virtual monoenergetic levels on coronary plaque volume components using photon-counting computed tomography. Eur. Radio..

[26] Oda S., Utsunomiya D., Nakaura T., Kidoh M., Funama Y., Tsujita K., Yamashita Y. (2019). Basic concepts of contrast injection protocols for coronary computed tomography angiography. Curr. Cardiol. Rev..

[27] Cundari G., Deilmann P., Mergen V., Ciric K., Eberhard M., Jungblut L., Alkadhi H., Higashigaito K. (2024). Saving contrast media in coronary CT angiography with photon-counting detector CT. Acad. Radio..

[28] Rajiah P.S., Dunning C.A.S., Rajendran K., Tandon Y.K., Ahmed Z., Larson N.B., Collins J.D., Thorne J., Williamson E., Fletcher J.G., McCollough C., Leng S. (2023). High-pitch multienergy coronary CT angiography in Dual-source photon-counting detector CT scanner at low iodinated contrast dose. Invest. Radio..

